# Explanatory Style in Asian Populations: A Scoping Review

**DOI:** 10.1192/j.eurpsy.2023.383

**Published:** 2023-07-19

**Authors:** C. Siu, E. Gacayan, V. W. L. Tsang

**Affiliations:** University of British Columbia, Vancouver, Canada

## Abstract

**Introduction:**

Cultural upbringing is an important factor that affects how one perceives the world and determines how blame is assigned when facing unfavorable outcomes. Governed by explanatory style, which refers to one’s pattern of causal explanations towards positive and negative situations, pessimism is defined as classifying bad events as internal, stable, and global within the personalization, permanence, and pervasiveness dimensions respectively (Peterson, 1992). This indicates that a pessimistic person would attribute a bad event as their own fault, as lasting, and as having great impact across all domains of their lives (Peterson, 1992).

**Objectives:**

Previous research has shown that when comparing the levels of pessimism between Mainland Chinese individuals, Chinese American individuals, and White American individuals, the Mainland Chinese group is found to be the most pessimistic (Lee & Seligman, 1997). However, much of the existing research to date assessing pessimism and optimism in Asian samples do not define these concepts within the realms of the explanatory style. Instead, a broader and more generalized understanding is usually used. This review therefore seeks to investigate whether Asians are more pessimistic compared to people of other races as defined by the explanatory style (Peterson, 1992).

**Methods:**

A search was conducted in two bibliographic databases (Medline OVID and PsycINFO) to identify articles for inclusion. Two reviewers screened the search independently through Covidence and performed a result analysis.

**Results:**

A total of 20 peer-reviewed articles published between 1972 and 2022 are included that broadly compare the explanatory styles of Asians to other racial groups. Of the 20 studies, 3 papers specifically compare optimism versus pessimism in Asian groups, 4 papers mention coping strategies for “bad events”, 2 papers mention self-esteem, and 2 papers each mention causal attributions for success and failure respectively. When compared to other races, Asians were found to be more pessimistic, turn to faith and religion as a coping mechanism, have lower self-esteem, attribute success to external factors and internalize failure.

**Conclusions:**

It is concluded that Asian groups internalize bad events, and view good events as external, which aligns with Asian groups being more pessimistic on the personalization dimension as defined by the explanatory style (Spencer-Rodgers et al., 2004; Anderson, 1999; Park & Kim, 1998). By understanding the cultural implications of the explanatory style, one can recognize why and in what way people behave and cope differently across cultures when facing adversity.

**Disclosure of Interest:**

None Declared

